# FREQUENCY OF CONTACT AMONG FAMILY MEMBERS AND END-OF-LIFE CARE PLANNING

**DOI:** 10.1093/geroni/igz038.2476

**Published:** 2019-11-08

**Authors:** Meghan McDarby, Elissa K Kozlov, Brian Carpenter

**Affiliations:** 1 Washington University in St. Louis, St. Louis, Missouri, United States; 2 Rutgers University, New Brunswick, New Jersey, United States

## Abstract

The purpose of the current analysis was to examine how contact between adult children and their older parents may relate to having end-of-life care conversations. We analyzed responses from adult children (n = 66) of 36 older adults (65+) who participated in an intervention to improve family communication. Children reported the frequency of their in-person and phone contact with parents. They also completed the Conversations about Care Arrangements Scale (alpha = 0.95), 8 items that measure the extent to which adult children have discussed plans about future care with their parents (1=have not talked at all, 5=talked extensively). We calculated a composite from all 8 items (potential range 5-40). Participants reported that they had, on average, not talked extensively with their parent about plans for future care (M = 18.7, SD = 8.22). Overall, 42.4% of children reported that they visited their parent one time or less per year. Children reported speaking on the phone with their parent an average of 4.74 times per week (SD = 6.80, range = 0-30) and initiating an average of 2.59 of those calls (SD = 3.82, range = 1-20). Frequency of weekly phone conversations between child and parent was significantly associated with having talked more extensively about future care plans (r = 0.25, p < 0.05), as was frequency of phone calls initiated by the adult child (r = 0.29, p < 0.05). Frequency of in-person visits to parents was not significantly associated with conversations. Infrequent contact may limit opportunities for care conversations.

